# Kinesiophobia as part of the psychological burden in Spondyloarthritis: a case–control study

**DOI:** 10.1093/rap/rkaf040

**Published:** 2025-04-11

**Authors:** Eugenio Capparelli, Maria Iacovantuono, Sergio Del Vescovo, Benedetta Monosi, Chiara Bonini, Luigi Fiannacca, Elisabetta Greco, Eneida Cela, Paola Conigliaro, Greta Giulia Dipietrangelo, Florenzo Iannone, Giuseppe Lopalco, Maria Sole Chimenti

**Affiliations:** Rheumatology, Allergology and Clinical Immunology, Department of Systems Medicine, University of Rome Tor Vergata, Rome, Italy; Rheumatology, Allergology and Clinical Immunology, Department of Systems Medicine, University of Rome Tor Vergata, Rome, Italy; Department of Precision and Regenerative Medicine and Ionian Area (DiMePRe-J), UNIBA, Bari, Italy; Rheumatology, Allergology and Clinical Immunology, Department of Systems Medicine, University of Rome Tor Vergata, Rome, Italy; Rheumatology, Allergology and Clinical Immunology, Department of Systems Medicine, University of Rome Tor Vergata, Rome, Italy; Rheumatology, Allergology and Clinical Immunology, Department of Systems Medicine, University of Rome Tor Vergata, Rome, Italy; Rheumatology, Allergology and Clinical Immunology, Department of Systems Medicine, University of Rome Tor Vergata, Rome, Italy; Rheumatology, Allergology and Clinical Immunology, Department of Systems Medicine, University of Rome Tor Vergata, Rome, Italy; Rheumatology, Allergology and Clinical Immunology, Department of Systems Medicine, University of Rome Tor Vergata, Rome, Italy; Department of Precision and Regenerative Medicine and Ionian Area (DiMePRe-J), UNIBA, Bari, Italy; Department of Precision and Regenerative Medicine and Ionian Area (DiMePRe-J), UNIBA, Bari, Italy; Department of Precision and Regenerative Medicine and Ionian Area (DiMePRe-J), UNIBA, Bari, Italy; Rheumatology, Allergology and Clinical Immunology, Department of Systems Medicine, University of Rome Tor Vergata, Rome, Italy

**Keywords:** spondyloarthritis, kinesiophobia, sex differences, psychological well-being, targeted interventions, biologic treatments

## Abstract

**Objectives:**

Psychological distress is commonly reported by patients affected by Spondyloarthritis (SpA), with >50% experiencing concomitant depression or anxiety. This case–control study aimed to investigate the psychological dimensions of SpA by assessing and comparing levels of kinesiophobia, depression and health-related quality of life (HRQoL) between SpA patients and a healthy control (HC) group.

**Methods:**

This cross-sectional case–control study included patients with SpA classified by Assessment of SpondyloArthritis international Society classification criteria and a group of HCs matched by sex and age. Inclusion criteria were age ≥18 years and stable therapy for at least 6 consecutive months. Data collection involved interviews and medical records. Psychological assessments were conducted using the Italian version of the Tampa Scale of Kinesiophobia-13 (TSK-IV), Beck’s Depression Inventory (BDI) and the 36-item Short Form (SF-36) Health Survey. Statistical analyses included *t*-test or Mann–Whitney U-test, chi-squared test, correlation analysis and multiple linear regression models.

**Results:**

Among 172 SpA patients and 94 HCs, SpA patients had significantly higher kinesiophobia (*P* < 0.001) and depression scores (*P* < 0.01). HRQoL was lower across all SF-36 domains except perceived health change. Axial SpA and peripheral SpA differed in diagnostic and therapeutic delay. Females showed greater kinesiophobia and depressive symptoms than males. Undergoing to second- or subsequent-line biologic therapy was linked to higher kinesiophobia and poorer HRQoL. BDI scores and diagnostic delay were key predictors of kinesiophobia in the SpA population.

**Conclusion:**

Kinesiophobia has a significant impact on psychological well-being in SpA patients. These findings highlight the need for targeted interventions that address not only the physical but also the psychological dimension of SpA.

Key messages

- Spondyloarthritis patients experience higher kinesiophobia and depression and reduced health-related quality of life compared with controls.
- Diagnostic delay and depressive symptoms are relevant predictors of kinesiophobia in spondyloarthritis.
- Targeted interventions are essential to address both the physical and psychological aspects of spondyloarthritis.

## Introduction

SpA refers to a group of inflammatory arthritides, primarily affecting the spine and peripheral joints [1]. Inflammatory back pain (IBP) and pain at entheseal sites are hallmark symptoms of the disease, making SpA a significant and compelling cause of chronic musculoskeletal pain [2].

Pain in SpA is complex and multifaceted, arising from inflammatory, mechanical and central sensitization mechanisms [3]. The inflammatory component, associated with cytokines such as TNF-α and IL-17, contributes to pain by promoting inflammation at typical sites, including the sacroiliac joints, spine and entheses [4]. The mechanical component of pain reflects the potential progression toward structural damage [5]. Additionally, SpA patients may experience neuropathic pain caused by lesions or diseases affecting the nervous somatosensory system [6].

Although achieving optimal control of inflammatory burden, limitations in spine and joint mobility often continue to negatively impact the health-related quality of life (HRQoL) in SpA patients [7]. Nevertheless, even in the absence of progressive structural damage, evidence suggests that in some patients with non-radiographic axial SpA (nr-axSpA), reduced disease activity does not lead to proportional improvements in physical function [8]. These inconsistencies indicate that additional factors, such as psychological components, may contribute to worsen the HRQoL [9].

In line with this psychological model, individuals inclined toward negative appraisal patterns, are more likely to perceive external stimuli—such as pain—as unpleasant, which in turn influences their cognitive and behavioural responses. In this context, pain catastrophizing and fear-avoidance behaviours have been identified as key contributors to central sensitization and the chronicity of pain [10]. Pain catastrophizing refers to the tendency to exaggerate the threat value of pain sensations, with affected individuals finding it difficult to divert their attention away from pain [11]. Additionally, the Fear-Avoidance Model explains how chronic musculoskeletal pain can lead to persistent avoidance beliefs [12]. In this context, individuals experiencing kinesiophobia, defined as the excessive and irrational fear of movement, perceive pain as harmful and may adopt maladaptive strategies, such as avoiding movement [13]. This can create a cycle in which increasing physical inactivity leads to muscular deconditioning and disability, ultimately intensifying pain sensations [14].

It was already established that kinesiophobia significantly impacts chronic pain–related disorders, such as OA, FM, RA and SLE [15, 16]. Although a limited number of studies have reported the prevalence of kinesiophobia in AS [17], there is still no evidence for patients with either ax-SpA [both radiographic ax-SpA (r-axSpA) and nr-axSpA] or peripheral SpA (p-SpA). Moreover, given that physical activity is included in treatment recommendations for SpA management [18], emerging evidence suggests that kinesiophobia may pose a significant barrier to effectiveness of treat-to-target strategies [19].

The aims of this study are to estimate the prevalence of kinesiophobia in patients with SpA compared with a healthy control (HC) group matched for age and sex; evaluate the relationship between depression, HRQoL and kinesiophobia; and investigate the impact of axial involvement, sex and refractoriness to biologic therapy on psychological outcomes in this population.

## Materials and methods

### Study design

This cross-sectional case–control study included patients recruited from two rheumatology units at Fondazione Policlinico Tor Vergata in Rome and the University Hospital Consortium Corporation Polyclinic of Bari. Each SpA patient received regular follow-up care within these hospitals. The enrolment period spanned from May 2023 to May 2024.

The inclusion criteria were patients affected by SpA and/or PsA according to Assessment of SpondyloArthritis international Society [20] and/or 2006 Classification Criteria for Psoriatic Arthritis [21], age ≥18 years and stable therapy (≥6 months) with at least one biologic DMARD (bDMARD) or targeted synthetic DMARD (tsDMARD). Exclusion criteria included recent episodes of musculoskeletal trauma or fractures, arthroplasty surgery and a concurrent diagnosis of FM (according to the 2016 Widespread Pain Index/Symptom Severity Scale EULAR diagnostic criteria) [22].

Written informed consent was obtained from each participant and the study was conducted in accordance with the ethical guidelines of the Declaration of Helsinki, with approval from the local ethics committee of Fondazione Policlinico Tor Vergata under protocol number 186/16.

### Data collection

Data were collected through structured interviews and medical records to gather information on participants’ demographic characteristics, including age, sex, smoking habits and educational status. Clinical data encompassed age at enrolment, disease duration and diagnostic and therapeutic delays (defined as the time interval, in months, from symptom onset to definitive diagnosis and initiation of biologic treatment). Main disease domains, including psoriasis (affecting skin and/or nails), dactylitis and peripheral enthesitis, were evaluated, along with the presence of non-infectious uveitis and inflammatory bowel disease as relevant SpA extra-articular manifestations. Comorbid conditions were carefully documented, with a particular focus on cardiovascular diseases, dyslipidaemia, type 2 diabetes mellitus, lung diseases and mental health issues such as anxiety and depression. Laboratory assessment included inflammatory markers such as CRP (0–5 mg/l) and ESR (0–30 mm/h).

To evaluate disease activity, several validated indices were employed, including the visual analogue scale for pain (VAS pain), the Patient Global Assessment (PtGA) and the Physician Global Assessment (PhGA). Additionally, tender and swollen joint counts (TJC68 and SJC66) were recorded at the last rheumatologic visit. The BASDAI, the Axial Spondyloarthritis Disease Activity Score based on CRP (ASDAS-CRP) and the Disease Activity Index for Psoriatic Arthritis (DAPSA) were used to quantify overall disease activity.

### Questionnaire submission

For this study, three validated assessment tools were used to evaluate both the psychological burden and HRQoL of participants: the13-item Tampa Scale of Kinesiophobia Italian validated version (TSK-IV) [23], the BDI [24] and the SF-36 [25]. The TSK was originally developed with 17 items to measure subjective levels of kinesiophobia using a 4-point Likert scale, ranging from ‘strongly disagree’ to ‘strongly agree’. However, in the Italian adaptation (TSK-IV), items 4, 8, 12 and 16—which were reverse-scored—were removed. This decision was based on findings from the cross-cultural adaptation and validation process, which showed that removing these items produced a two-factor structure that optimized both variance and clinical relevance. The final TSK-IV consists of 13 items, grouped into two subscales: TSK-1 (items 1, 2, 10, 14, 15 and 17), which refers to the fear of reinjury, and TSK-2 (items 3, 5, 6, 7, 9, 11 and 13), which addresses beliefs about the harmfulness of physical activity. Scores on the TSK-IV range from 13 to 52, with higher scores indicating higher kinesiophobia. The BDI is a widely recognized 21-item self-reported questionnaire designed to assess the severity of depressive symptoms. Each item is scored from 0 to 3, with higher total scores reflecting more severe depression. The SF-36 is a comprehensive tool for evaluating HRQoL across eight domains, including physical functioning, emotional well-being and social role functioning. Responses are scored from 0 to 100, with higher scores indicating better health status. Percentage scores for the SF-36 were calculated using online tools [26]. Similarly, TSK-IV, BDI and SF-36 questionnaires were administered to HCs via an online form. Controls were also asked to provide their age, sex, height and weight to ensure appropriate matching with the patient group.

### Statistical analysis

The patients’ data were summarized as mean and s.d. for normally distributed variables or as median and interquartile range (IQR) for skewed ones. Discrete or qualitative variables were summarized as counts and percentages. Statistical inferential analysis was conducted to compare multiple variables. Mean differences of continuous variables were assessed using Student’s *t*-test or Mann–Whitney U-test, depending on whether the data followed a parametric or non-parametric distribution. Chi-squared and Fisher’s exact tests were used to compare categorical variables, depending on the sample size. Correlation analysis was performed by calculating Pearson’s or Spearman’s correlation coefficient, depending on the data distribution. Multivariate analysis was conducted using linear regression models for continuous independent variables. Bonferroni–Holm correction was applied to report multiple comparisons.

The total TSK-IV, as indicative of kinesiophobia levels across the SpA population, was selected as the dependent variable in the multiple linear regression analysis. The multiple linear regression model included several potential contributors (e.g. BASDAI, ASDAS-CRP, DAPSA, age at enrolment, sex, BMI, BDI scores, disease duration and diagnostic delay) that were identified based on previous literature data and after correlation analysis. A *P*-value ≤0.05 or a 95% confidence interval (CI) not crossing zero were considered statistically significant. Statistical analysis was conducted via SPSS Statistics version 27 (IBM, Armonk, NY, USA).

## Results

### The case–control study

**Figure 1.**
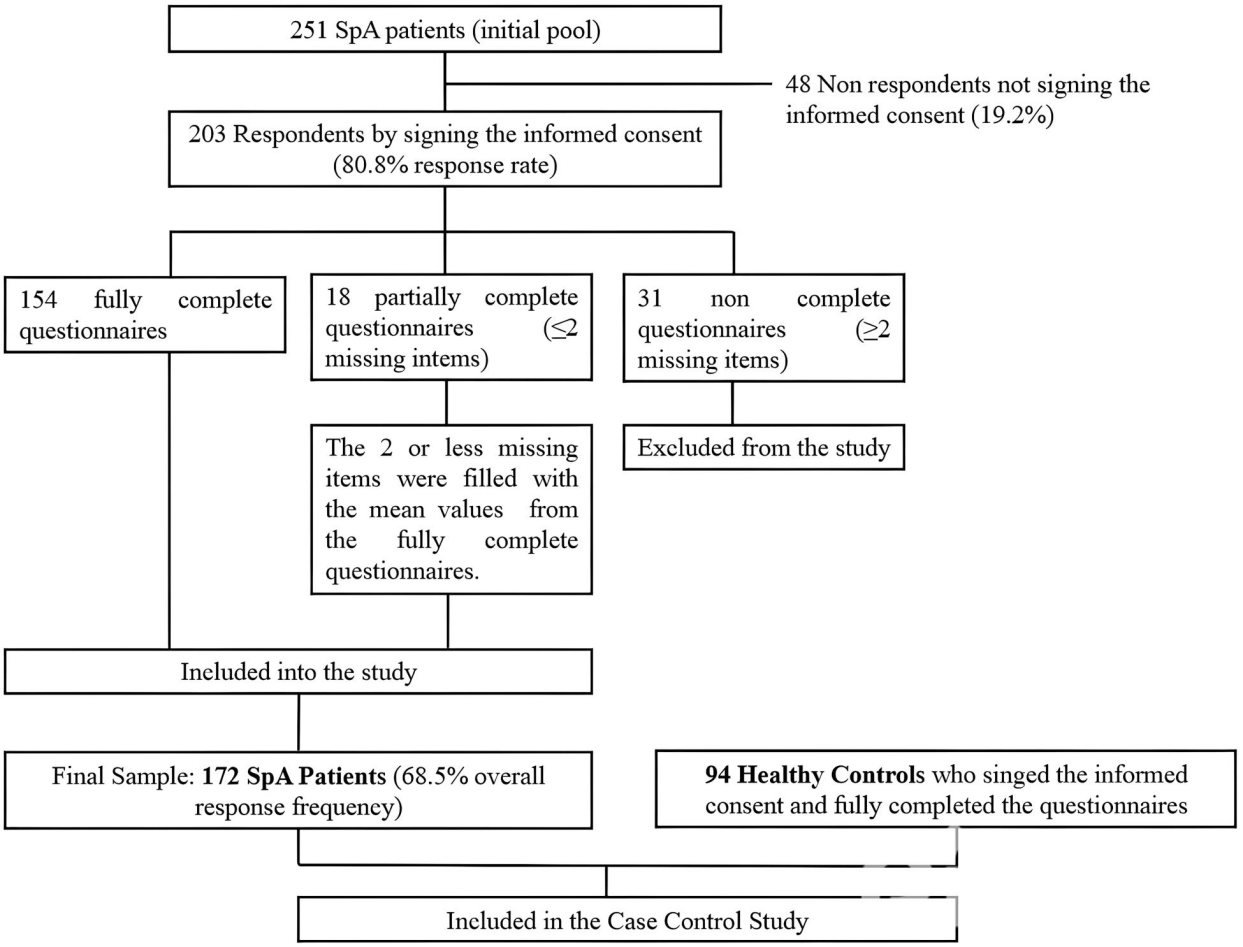
Flow chart on the final sample selection and questionnaire completion. A total of 203 of 251 (80.8%) patients returned the questionnaires and signed the informed consent to participate in the study. Of these, 154 participants provided fully complete questionnaires. An additional 18 questionnaires, with two or fewer missing items, were included by filling in the missing responses with the mean values from the fully complete questionnaires. The remaining questionnaires were excluded due to having more than two missing items, leading to a final sample of 172 questionnaires and an overall response rate of 68.5%

The final sample selection and questionnaire completion is reported in Fig. 1. The comparative study matching patients with SpA (*n* = 172) to HCs (*n* = 94) revealed no significant age and sex differences between the groups, although SpA patients had a notably higher BMI than controls [26.1 kg/m^2^ (s.d. 5.1) *vs* 24.3 (s.d. 3.9); *P* = 0.0032]. SpA patients exhibited significantly higher scores on the TSK-IV scale compared with HCs [28.4 (s.d. 9.7) *vs* 22.9 (s.d. 7.3); *P* = 0.0001]. Both subscales, TSK-1 and TSK-2, were elevated among SpA patients compared with HCs [12.2 (s.d. 4.6) *vs* 9.6 (s.d. 3.1); *P* = 0.0001 for TSK-1; and 15.6 (s.d. 4.9) *vs* 13.4 (s.d. 4.8); *P* = 0.0005 for TSK-2]. Additionally, SpA patients had higher BDI scores [8.4 (s.d. 7.3) *vs* 5.9 (s.d. 6.1); *P* = 0.0051] and reported significantly worse outcomes across almost all domains of the SF-36 questionnaire compared with HCs [Table 1].

**Table 1.** Kinesiophobia, depression and QoL indicators between cases and controls

Indicators	SpA (*n* = 172)	HCs (*n* = 94)	Effect size(95% CI)	*P*-value
Female, *n* (%)	97 (56.4)	56 (59.6)	0.0058 (0.0019, 0.137)	0.6160
Age at enrolment, years, mean (s.d.)	53.5 (10.3)	51.7 (12.5)	0.162 (−0.090, 0.414)	0.2083
BMI, kg/m^2^, mean (S.D.)	26.1 (5.1)	24.3 (3.9)	0.382 (0.128, 0.635)	**0.0032**
Smokers, *n* (%)	55 (32)	33 (35.1)	0.00137 (0.0019, 0.137)	0.6041
TSK, mean (S.D.)	28.4 (9.7)	22.9 (7.3)	0.616 (0.359, 0.873)	**0.0001**
TSK-1, fear of movement, mean (S.D.)	12.2 (4.6)	9.6 (3.1)	0.629 (0.372, 0.886)	**0.0001**
TSK-2, pain as cause of reinjury, mean (S.D.)	15.6 (4.9)	13.4 (4.8)	0.452 (0.198, 0.707)	**0.0005**
BDI, mean (S.D.)	8.4 (7.3)	5.9 (6.1)	0.362 (0.109, 0.616)	**0.0051**
SF-36, mean (S.D.)				
Physical functioning	62.7 (28.8)	91.9 (13.1)	−1.194 (−1.465, −0.923)	**0.0001**
Physical health	45.9 (41.9)	89.1 (25.1)	−1.145 (−1.414, −0.875)	**0.0001**
Emotional problems	45.6 (41.6)	83.7 (32.7)	−0.985 (−1.249, −0.720)	**0.0001**
Energy/fatigue	50.2 (20.0)	57.5 (18.1)	−0.377 (−0.631, −0.124)	**0.0036**
Emotional well-being	61.4 (16.6)	67.1 (17.3)	−0.338 (−0.591, −0.085)	**0.0089**
Social functioning	59.0 (22.3)	72.2 (21.8)	−0.597 (−0.853, −0.340)	**0.0001**
Pain	51.7 (24.9)	83.9 (20.5)	−1.373 (−1.651, −1.096)	**0.0001**
General health	42.9 (18.6)	64.5 (15.6)	−1.227 (−1.499, −0.955)	**0.0001**
Health change	50.9 (22.2)	53.7 (20.1)	−0.130 (−0.382, −0.121)	0.3105

Significant values in bold.

Effect size and 95% CI were calculated through Hedges’ *g* formula for unpaired *t*-tests and through V-Cramer for chi-squared analysis.

### Comparative analysis among intergroups

A further comparative analysis was conducted in the entire SpA population by selecting peripheral *vs* axial involvement, sex and refractoriness to first-line biologic therapy as compelling variables [Table 2]. Among the study population, 59 patients (34.3%) had ax-SpA (patients with r-axSpA were 44.1%, while patients with nr-axSpA were 55.9%), while 113 patients (65.7%) had p-SpA. A total of 92 patients (53.5%) had a diagnosis of PsA. There was a statistically significant difference in the prevalence of PsA in the p-SpA group compared with the ax-SpA group (*P* < 0.001). Additionally, females were more frequently represented in the p-SpA group than the ax-SpA group (*P* < 0.01). The diagnostic delay was longer for ax-SpA patients, with an average delay of 48.2 months (s.d. 57.2) compared with 27.4 months (s.d. 26.6) for p-SpA, even without reaching statistical significance. Conversely, the therapeutic delay was notably shorter in the ax-SpA group compared with the p-SpA group [9.0 months (s.d. 3.6) *vs* 31.3 (s.d. 30.7); *P* < 0.001]. In terms of disease manifestations, skin and nail psoriasis, peripheral enthesitis and dactylitis were more frequently observed in patients with p-SpA compared with ax-SpA, although only dactylitis reached a statistic significance (*P* < 0.05). No significant differences were found between the groups regarding other comorbidities and lines of b/tsDMARD treatments.

**Table 2.** Demographic, clinical and disease characteristics of the overall patient cohort across different intergroups.

Characteristics	Total	ax-SpA	p-SpA	Male	Female	First-line bDMARDs	≥Second-line bDMARDs
Patients, *n* (%)	172 (100)	59 (34.3)	113 (65.7)	75 (43.6)	97 (56.4)	90 (52.3)	82 (47.7)
Peripheral involvement, *n* (%)	113 (65.7)		113 (100)	**41 (54.7)** **	**72 (74.2)** **	57 (63.3)	56 (68.3)
nr-axSpA, *n* (%)	33 (19.2)	33 (55.9)		15 (20)	18 (18.6)	19 (21.1)	14 (17.1)
r-axSpA, *n* (%)	26 (15.1)	26 (44.1)		14 (18.7)	12 (12.4)	13 (14.4)	13 (15.9)
PsA, *n* (%)	92 (53.5)	**17(28.8)** ***	**75 (66.4)** ***	33 (44)	59 (60.8)	41 (45.6)	51 (62.2)
Female, *n* (%)	97 (56.4)	**25 (42.4)** *	**72 (63.7)** *			44 (48.9)	53 (64.6)
Age at enrolment, years, mean (s.d.)	53.3 (10.3)	53.5 (8.8)	53.5 (11.1)	54.4 (10.3)	52.8 (10.3)	52.5 (11.5)	54.6 (8.8)
Age at SpA diagnosis, years, mean (S.D.)	44.1 (12.2)	42.7 (11.8)	43.6 (13.2)	42.8 (12.0)	43.6 (12.3)	43.5 (13.3)	43.0 (12.1)
Disease duration, months, mean (S.D.)	118.0 (100.3)	131.4 (105.6)	126.9 (103.3)	133.6 (103.7)	124.5 (103.5)	**105.9 (85.4)** **	**153.2 (116.3)** **
Diagnostic delay, months, mean (S.D.)	35.1 (43.6)	48.2 (57.2)	27.4 (26.6)	36.9 (55.2)	24.0 (31.5)	**26.3 (22.2)** *	**43.0 (52.4)** *
Therapeutic delay, months, mean (S.D.)	26.9 (28.8)	**9.0 (3.6)** ***	**31.3 (30.7)** ***	27.5 (35.6)	26.2 (22.1)	14.9 (15.4)	34.8 (33.4)
BMI, kg/m^2^, mean (S.D.)	26.0 (4.9)	27.6 (5.1)	26.6 (5.1)	27.7 (4.6)	26.3 (5.4)	26.4 (4.9)	27.5 (5.3)
Obese (BMI ≥30 kg/m^2^), *n* (%)	39 (22.7)	18 (30.5)	21 (18.8)	18 (24.3)	21 (21.6)	**15 (16.7)** *	**24 (29.6)** *
Smoker, *n* (%)	55 (32)	19 (32.8)	36 (31.9)	27 (36)	28 (28.9)	33 (36.7)	22 (26.8)
Education level, *n* (%)							
Compulsory school	57 (33.1)	23 (39)	34 (30.1)	23 (30.7)	34 (35.1)	**22 (24.4)** *	**35 (42.7)** *
Diploma	75 (43.6)	22 (37.3)	53 (46.9)	34 (45.3)	41 (42.3)	44 (48.9)	31 (37.8)
Degree	40 (23.3)	14 (23.7)	26 (23)	18 (24.0)	22 (22.7)	24 (26.7)	16 (19.5)
Disease domains, *n* (%)							
Cutaneous psoriasis	99 (57.6)	26 (44.1)	73 (64.6)	41 (54.7)	58 (59.8)	46 (51.1)	53 (64.6)
Nail psoriasis	38 (22.1)	7 (11.9)	31 (27.4)	12 (16)	26 (26.8)	18 (20)	20 (24.4)
Dactylitis	39 (22.7)	**6 (10.2)** *	**33 (29.2)** *	17 (22.7)	22 (22.7)	18 (20)	21 (25.6)
Peripheral enthesitis	93 (54.1)	25 (42.4)	68 (60.2)	43 (57.3)	50 (51.5)	47 (52.2)	46 (56.1)
Non-infectious uveitis	13 (7.6)	6 (10.2)	7 (6.2)	4 (5.3)	9 (9.3)	5 (5.6)	8 (9.8)
IBD	14 (8.1)	6 (10.2)	8 (7.1)	8 (10.7)	6 (6.2)	7 (7.8)	7 (8.5)
Comorbidities, *n* (%)							
Cardiovascular diseases	60 (34.9)	18 (30.5)	42 (37.2)	32 (42.7)	28 (28.9)	26 (28.9)	34 (41.5)
Dyslipidaemia	47 (27.3)	18 (30.5)	29 (25.7)	19 (25.3)	28 (28.9)	19 (21.1)	28 (34.1)
Neoplasm	5 (2.9)	3 (5.1)	2 (1.8)	2 (2.7)	3 (3.1)	2 (2.2)	3 (3.7)
Pulmonary comorbidity	15 (8.8)	8 (13.6)	7 (6.2)	8 (10.7)	7 (7.2)	4 (4.4)	11 (13.4)
Type 2 diabetes mellitus	17 (9.9)	8 (13.6)	9 (8)	6 (8)	11 (11.3)	5 (5.6)	12 (14.6)
Anxiety/depression	22 (12.8)	9 (15.3)	13 (11.5)	7 (9.3)	15 (15.5)	8 (8.9)	14 (17.1)
bDMARD/tsDMARD treatment, *n* (%)							
First line	90 (52.3)	33 (55.9)	57 (50.4)	46 (61.3)	44 (45.0)		
Second line or superior	82 (57.7)	26 (44.1)	56 (49.6)	29 (38.7)	53 (54.6)		

Significant values in bold.

* *P* < 0.05,

** *P* < 0.01,

*** *P* < 0.001.

A sex-based analysis, including 75 males (43.6%) and 97 females (56.4%), indicated that therapeutic delay and disease duration were similar across the sexes. Peripheral involvement was more common in females (*P* < 0.01). Educational attainment did not differ significantly between the sexes, however, a higher percentage of females only completed compulsory schooling while more males held a diploma. Analysis of disease domains showed no significant sex differences. In terms of comorbidities, males tended to have a higher prevalence of cardiovascular diseases compared with females.

By comparing SpA patients undergoing first-line bDMARD therapy with those receiving second-line or superior bDMARD therapy, the analysis revealed that 45.4% of females were in the first-line therapy group while 54.6% were in the second-line group. Additionally, the prevalence of PsA was 45.6% in the first-line group, increasing to 62.2% in the second-line group. Obesity was present in 16.7% of patients in the first-line group, increasing to 29.6% in the second-line group (*P* < 0.05). Diagnostic delay averaged 26.3 months (s.d. 22.2) in the first-line group compared with 43.0 months (s.d. 52.4) in the second-line group (*P* < 0.05). As expected, disease duration was longer in the second-line group compared with the first-line group (*P* < 0.01).

### Disease outcome measures across intergroups

**Figure 2.**
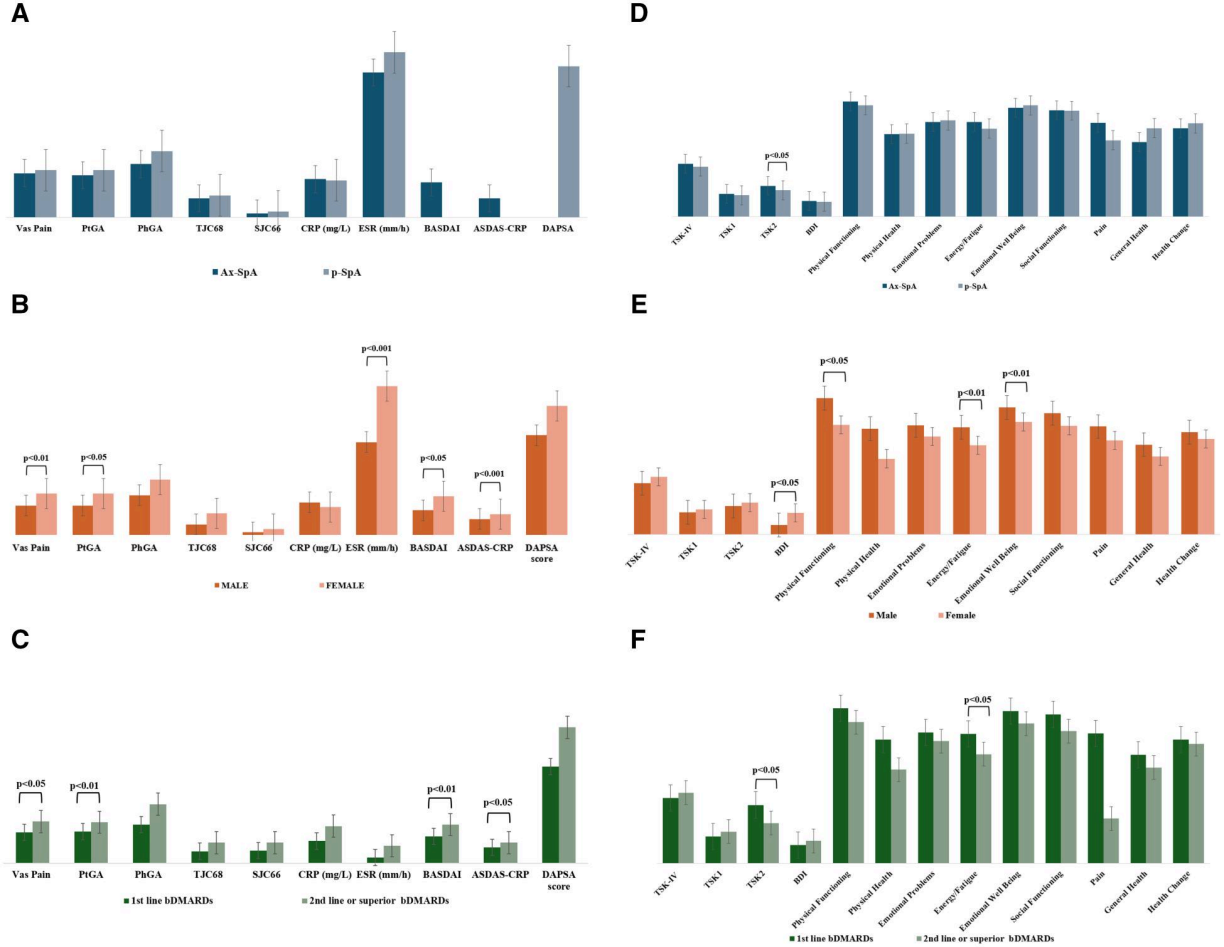
Disease outcome measures and comparative analysis of TSK, BDI and SF-36 scores across intergroups. **(A)** Disease outcome measures between ax-SpA and p-SpA. **(B)** Disease outcome measures between male and female patients. **(C)** Disease outcome measures between first-line bDMARD therapy and second-line or superior bDMARD therapy. **(D)** Comparative analysis of kinesiophobia, BDI and SF-36 scores between ax-SpA and p-SpA. **(E)** Comparative analysis of kinesiophobia, BDI and SF-36 scores between male and female patients. **(F)** Comparative analysis of kinesiophobia, BDI and SF-36 scores between first-line bDMARD therapy and second-line or superior bDMARD therapy

Significant differences were observed according to sex and treatment line, with females and patients on second-line or superior bDMARD therapies reporting higher VAS pain scores (*P* < 0.01 for both) and PtGA scores (*P* < 0.001 for female and *P* < 0.05 for second-line or superior bDMARDs group). Additionally, ESR was higher in females compared with males [18.8 mm/h (s.d. 18.8) *vs* 11.7 (s.d. 9.4); *P* < 0.05) regardless of CRP values, which showed no differences between the sexes. Composite disease activity outcomes were significantly elevated in females and in the second-line therapy group (*P* < 0.001 for BASDAI for females and *P* < 0.01 for second-line bDMARDs group; *P* < 0.05 for ASDAS-CRP for both groups). DAPSA scores showed no statistical differences across intergroups (Fig. 2).

### Kinesiophobia, depression and HRQoL across intergroups

As shown in Fig. 2, TSK-IV results indicated no significant differences in overall kinesiophobia between ax-SpA and p-SpA or between r-axSpA and nr-axSpA. However, ax-SpA patients reported a significantly higher mean score in the TSK-2 subscale (*P* < 0.05). Similarly, TSK-2 subscale scores were greater in the second-line bDMARDs therapy group (*P* < 0.05). However, even without reaching statistical significance, the total TSK score was higher in females compared with males [29.8 (s.d. 9.4) *vs* 26.5 (s.d. 9.9)]. The BDI scores were higher in females compared with males (*P* < 0.01). HRQoL revealed significant sex differences in multiple domains. Similarly, significant discrepancies were found in several SF-36 domains for energy/fatigue for the second-line bDMARDs therapy group compared with the first-line therapy group (*P* < 0.05). Additionally, the TSK-IV total score and TSK-1 and TSK-2 subscales were evaluated by comparing patients with a known diagnosis of depression and/or anxiety and those without. The TSK-IV total score and TSK-2 differed significantly [36 (s.d. 10.3) *vs* 28 (s.d. 9.2); *P* < 0.001 for TSK-IV; and 20 (s.d. 5.6) *vs* 15 (s.d. 4.7); *P* = 0.011 for TSK-2], while TSK-1 scores did not differ between the two groups.

### Correlation analysis and multiple linear regression analysis

**Figure 3.**
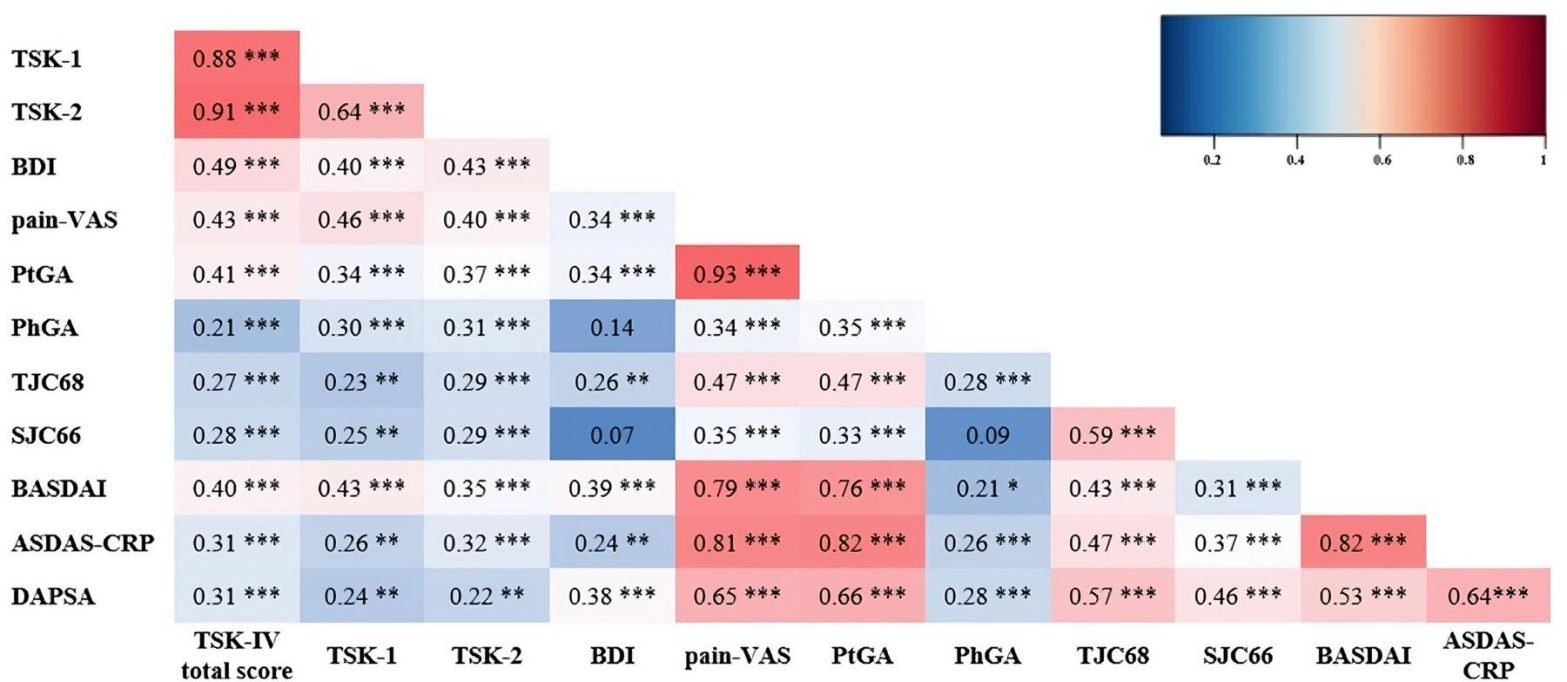
Heat map showing correlations among TSK-IV, TSK-1 and TSK-2, BDI and disease outcome measures among the SpA population. *P < 0.05; **P < 0.01; ***P < 0.001

As shown in Fig. 3, several correlations emerged between kinesiophobia and various clinical parameters in patients with SpA. The TSK-IV total score scale, TSK-1 and TSK-2 were positively correlated with all disease outcome measures (VAS pain, PtGA, PhGA, TJC68, SJC66, BASDAI, ASDAS-CRP and DAPSA). In contrast, BDI did not show a correlation with PhGA and SJC66. The correlation between TSK-IV total score and the BASDAI was stronger (*r* = 0.40, *P* < 0.001) than that with ASDAS-CRP and DAPSA (*r* = 0.31, *P* < 0.001 for both). Similar results emerged correlating TSK-1 with the BASDAI (*r* = 0.43, *P* < 0.001), ASDAS-CRP (*r* = 0.26, *P* < 0.01) and DAPSA (*r* = 0.24, *P* < 0.01). On the other hand, the correlation between TSK-2 and the BASDAI (*r* = 0.35, *P* < 0.001) and ASDAS-CRP (*r* = 0.32, *P* < 0.001) was stronger than that with DAPSA (*r* = 0.22, *P* < 0.01). Interestingly, moderate correlations emerged between depression levels, as reported by BDI scores and the TSK-IV total score (*r* = 0.49, *P* < 0.001) and TSK-1 (*r* = 0.40, *P* < 0.001) and TSK-2 subscales (*r* = 0.43, *P* < 0.001).

The multiple linear regression model showed that longer diagnostic delay (β = 0.05055, *P* = 0.007) and greater BDI scores (β = 0.36702, *P* = 0.003) were predictors of increased kinesiophobia in our population, highlighting the compounded impact of diagnostic delay and depressive symptoms on fear of movement [Table 3].

**Table 3.** Multiple linear regression analysis

	Beta coefficient	Standard error	95% CI	*p*-value
TSK-IV total score (intercept)	33.32925	5.72366	21.88781, 44.7707	<0.001
Age at enrolment	−0.03415	0.08244	−0.19895, 0.1306	0.680
Female sex	−1.51083	1.80242	−5.11382, 2.0922	0.405
BMI	−0.17480	0.16845	−0.51153, 0.1619	0.303
Disease duration	0.00587	0.00782	−0.00977, 0.0215	0.456
Diagnostic delay	0.05055	0.01822	0.01413, 0.0870	0.007
ASDAS-CRP	−0.84650	1.32080	−3.4875, 1.7937	0.524
BASDAI	0.53978	0.53498	−0.52963, 1.6092	0.317
DAPSA	0.00047	0.06387	−0.12720, 0.1281	0.994
BDI	0.36702	0.11958	0.12798, 0.6061	0.003

TSK-IV total score was used as a dependent variable as indicative of kinesiophobia levels in the study cohort.

Variables that were significantly correlated with TSK-IV total scores and/or clinically relevant were selected as independent variables for the model. Age at enrolment, sex, BASDAI, ASDAS-CRP, DAPSA, BMI, disease duration, diagnostic delay and BDI scores were included.

## Discussion

The present study is the first investigating the prevalence of kinesiophobia in patients with ax-SpA and p-SpA. A notable finding was the elevated levels of kinesiophobia in this population compared with an HC group, as evidenced by higher scores on the TSK-IV scale. This is in line with previous research showing that individuals with chronic pain conditions often develop fear-based beliefs regarding movement [27]. Specifically, the TSK-2 subscale, which measures beliefs about the harmfulness of physical activity, was particularly elevated in SpA patients, indicating a significant fear of practicing exercise that can worsen their pain-related condition. This is critical, as kinesiophobia can lead to a cycle of avoidance behaviours that restrict mobility and exacerbate functional limitations, sedentary lifestyle and disability [28]. Remarkably, our study revealed a moderate correlation between kinesiophobia—TSK-IV total score and TSK-1 and TSK-2 subscales—and pain VAS, suggesting that pain could significantly contribute to kinesiophobia in this population. Accordingly, previous evidence by Lööf *et al.* [29] established the presence of pain-related avoidance behaviour in other inflammatory arthritis, such as RA, emphasizing the importance of cognitive behavioural therapy. A similar relationship has been observed in patients with AS, chronic low back pain and SLE [16]. Our study showed a stronger correlation between TSK-IV scores and the BASDAI than between TSK-IV and ASDAS-CRP scores and DAPSA. This suggests that although ASDAS-CRP and DAPSA effectively measure disease activity by including CRP in their calculation, they may not fully capture the psychological factors affecting SpA patients when reporting disease activity [30]. Interestingly, our results highlight the intricate relationship between mental health and physical condition in SpA patients, as reported by elevated BDI scores in the SpA population compared with controls and as shown by the correlation between BDI scores and the TSK scale and subscales. Moreover, SpA patients with a concomitant diagnosis of depression/anxiety exhibited worse scores only in the TSK-IV and TSK-2 scales, indicating a greater perception of pain as a cause of reinjury. These data demonstrate that, even in the absence of a definite diagnosis of mental disturbance, the fear of movement could be present in this population, as shown by similar scores in the TSK-1 subscale.

Individuals extensively focusing on their pain condition may be more susceptible to mood disorders, which can, in turn, lower the pain threshold and amplify symptoms, leading to further avoidant behaviours. Subsequently, depression can intensify pain perception and trigger central pain sensitization, leading to FM [31, 32]. Interestingly, patients with FM were excluded from our analysis, providing a valuable perspective.

It is well-documented that FM patients often experience high levels of pain catastrophizing and kinesiophobia [33]. However, our data suggest that even in the absence of a definite diagnosis of FM, SpA patients may exhibit movement avoidance behaviours, underscoring the role of both mechanical and psychological factors in SpA [34]. As expected, the substantial negative correlation between kinesiophobia and HRQoL emphasizes the impact on overall health of these maladaptive behavioural strategies [35].

Our study also reports valuable insights through different intergroup comparisons, particularly regarding the sociocultural and psychological dimensions of SpA. For instance, educational attainment differed between the sexes even without statistical significance, with males tending to have a higher proportion of diploma-level education than females. This observation aligns with findings by Stonkute *et al.* [36], who reported larger educational inequalities in disability-free life expectancy among women in Central and Eastern Europe. Additionally, the analysis revealed several disparities in disease outcomes between the sexes, with females reporting higher scores in VAS pain and PtGA and higher levels of ESR, along with greater scores on the BASDAI and ASDAS-CRP for axial forms. As shown in previous literature, females diagnosed with axSpA tend to have lower treatment adherence and a reduced response to therapy, experiencing greater limitations in physical function despite showing less structural spinal damage compared with males [37]. Valuable insights have emerged concerning kinesiophobia, depression and HRQoL across intergroups analysis. First, females experienced higher levels of depressive symptoms and poorer HRQoL across several SF-36 domains compared with males. Existing evidence indicates that females with chronic diseases often report worse psychological scores than males [38]. Additionally, the significant increase in kinesiophobia among those undergoing second-line therapies, as reported by higher scores on the TSK-2 subscale, suggests a potential confounding influence of psychological factors on disease activity measures, regardless of the underlying inflammatory process. This finding highlights the need to incorporate psychological support into management strategies for SpA patients experiencing primary bDMARD failure [39].

The comparative analysis revealed statistical differences in terms of diagnostic delay for the second-line bDMARD therapy group, while a trend toward significance was observed for the ax-SpA group compared with the p-SpA group. Despite recent evidence suggesting improvements in diagnostic delays in ax-SpA over past decades, the timeframe for diagnosis remains unacceptably long, with an average delay of 6.7 years worldwide [40]. Meanwhile, a recent multicentre nationwide analysis reported a median diagnostic delay of 35.1 months in PsA, with PsA patients still facing a 2-fold risk of delayed diagnosis compared with RA [41]. This challenge is compounded by the absence of specific disease biomarkers, such as the lack of disease-characterizing autoantibodies [41], along with the lower levels of education, arthritis symptoms preceding skin manifestations and low back pain at the first visit [42].

These findings align with our results and underscore the heterogeneity of SpA symptoms, which may hinder prompt diagnosis, particularly in patients with lower educational attainment, axial involvement and no personal or familial history of psoriasis. In fact, there is still limited evidence regarding the actual diagnostic delay in patients with p-SpA without psoriasis. Our study provides valuable data in this area, reporting a median diagnostic delay of 30.2 months (s.d. 29.6) for p-SpA, compared with a median delay of 25.9 months (s.d. 25.0) in PsA. These results highlight the ongoing challenges in early recognition and diagnosis of both the peripheral and axial forms of SpA, especially in the absence of psoriasis. Furthermore, diagnostic delay seems to play a pivotal role in exacerbating kinesiophobia in SpA patients, as emerged in the multiple linear regression model, suggesting that prolonged waiting for a clear diagnosis could intensify patients’ fears related to movement. This finding underscores the critical need for early diagnosis in SpA, which, as previously suggested, significantly enhances treatment effectiveness and the likelihood of remission and, as we demonstrated, could help to mitigate fears related to physical activity [43]. In contrast, the delay in initiating biologic therapy was significantly shorter in ax-SpA than p-SpA patients. This finding aligns with recent SpA treatment guidelines that recommend bDMARDs as a first-line therapy after 1 month of unsuccessful NSAID treatment for ax-SpA. However, for p-SpA, the guidelines strongly recommend the initial use of csDMARDs, such as methotrexate [19].

While this study provides valuable insights, it has several limitations. The cross-sectional design restrained the causal inferences regarding the relationship between kinesiophobia, psychological health and disease outcomes. Longitudinal studies are warranted to assess how these factors evolve over time and impact disease progression and treatment responses. Additionally, as the study was conducted in two Italian centres, the generalizability of the findings may be limited. Future research should consider multicentre and international collaborations to enhance diversity in study populations.

## Conclusion

This study highlights the multifaceted nature of SpA, demonstrating a higher prevalence of kinesiophobia and depression and poorer HRQoL among these patients. Future research should aim to further clarify these relationships and guide tailored interventions, prioritizing early diagnosis and psychological support to improve disease outcomes, physical function and overall well-being of individuals with SpA.

## Data Availability

The data that support the findings of this study are available from the corresponding author upon reasonable request.
